# Reporting of Differences in Taste Between Branded and Unbranded Cigarettes by Smokers Blinded to Cigarette Branding: Within-Person, Randomized Crossover Study

**DOI:** 10.2196/24446

**Published:** 2021-05-14

**Authors:** Nasser F BinDhim, Nora A Althumiri, Mada H Basyouni, Rasha A Almubark, Zaied Alkhamaali, Weam Banjar, Mohammed Zamakhshary, Khaled M AlKattan

**Affiliations:** 1 Sharik Association for Health Research Riyadh Saudi Arabia; 2 College of Medicine Alfaisal University Riyadh Saudi Arabia; 3 Saudi Food and Drug Authority Riyadh Saudi Arabia; 4 Ministry of Health, Saudi Arabia Riyadh Saudi Arabia

**Keywords:** smoking, plain packaging, sensory, Saudi Arabia, tobacco, virtual reality, cigarettes

## Abstract

**Background:**

Saudi Arabia implemented a plain tobacco packaging regulation, one of the World Health Organization’s recommended initiatives to help reduce smoking rates, in August 2019. A few weeks after implementation, a large number of smokers complained via various media channels, especially social media (eg, Twitter), that an extreme change in cigarette taste had occurred, frequency of coughing had increased, and for some, shortness of breath had led to hospitalization.

**Objective:**

The main objective is to determine whether smokers blinded to cigarette branding report differences in taste between branded and unbranded cigarettes. The secondary objective is to observe the frequency of immediate cough or shortness of breath.

**Methods:**

This study employed a within-person, randomized crossover design that recruited current smokers 18 years and older who were cleared upon physical assessment before the experiment. Participants received 6 sequences of different random exposures (3 puffs) to 3 plain-packaged cigarettes (2 from their favorite brand and 1 from another brand as a control) and 3 branded cigarettes (2 from the favorite brand and 1 from another brand as a control). Participants wore virtual reality goggles accompanied by special software to alter visual reality and gloves to alter the touch sensation.

**Results:**

This study recruited 18 participants, measured at 6 time points, to produce 108 experiments. Participants were not able to identify the correct type of cigarettes (plain or branded, estimate of fixed effect=−0.01, *P*=.79). Moreover, there were no differences in the ability of the participants to identify their favorite brand (*t*_107_=−0.63, mean 0.47, *P*=.53). In terms of immediate coughing, out of the 108 experiments, 1 episode of short coughing was observed, which was attributed to the branded cigarette, not the plain-packaged cigarette.

**Conclusions:**

After controlling the visual and touch sensations, participants were not able to differentiate between branded and plain-packaged cigarettes in terms of taste or inducing immediate shortness of breath or cough. Interestingly, participants were not able to identify their favorite brand.

## Introduction

### Overview

The prevalence of cigarette smoking among adults in Saudi Arabia is between 16.1% and 21.1% [1-3]. Saudi Arabia implemented a plain tobacco packaging regulation in late August 2019. A few weeks after implementation, a huge number of smokers complained via various media channels (especially social media) that an extreme change in cigarette taste had occurred, frequency of coughing had increased, and for some, shortness of breath had led to hospitalization. The taste claims have arisen in other countries that have implemented plain packaging, such as Australia and the United Kingdom [4-6].

These complaints persisted for more than 90 days, starting in mid-November 2019 and continuing until the writing of the first draft of this manuscript, on March 2020. Rumors that the cigarettes currently sold in Saudi Arabia in plain packaging have toxic chemicals or other non-tobacco substances intended to harm smokers are circulating widely in the media and by word of mouth [7,8].

Saudi authorities requested that tobacco companies declare any changes of their cigarette content beyond the new plain-packaging requirements. British American Tobacco Middle East and Philip Morris International released public statements declaring they had made no changes to their products’ contents (Multimedia Appendix 1) [6].

Nevertheless, citizens and visitors in Saudi Arabia have been anxious and concerned about the health consequences of consuming the current plain-packaged cigarettes [7]. Consequently, prices of branded cigarettes tripled, and smuggling increased dramatically [7,8].

The results of chemical analysis and examination of conformity with Saudi tobacco product and safety standards have shown the new plain-packaged cigarettes are within the standards, and no unusual level of toxicity was found [7]. However, the claims made in the media about the taste, immediate coughing, and shortness of breath have not been investigated.

A few studies have investigated this issue in other countries that implemented plain packaging [9-11]. These studies did not find significant differences in taste, but they highlighted the difficulties of measuring this variable, which may affect the results. The main difficulty is in the method of measuring the difference between the branded and the plain-packaged cigarettes without exposing participants to the brand they are trying during the study. No previous study was fully able to blind the participants to the cigarette branding, although the senses are known to affect the taste.

### How Does Changing Vision and Touch Senses Affect Taste?

The stimulation of one sense organ influences, to some extent, the sensitivity of organs of another sense [12]. Human beings have 5 senses that are each interlinked. Changing or altering the vision or touch senses affects taste. In fact, taste involves a combination of gustatory and olfactory stimuli [13]. Although vision is not directly related to taste, changing vision alters the perception that a person might have about something, which leads to a change in its taste [14]. When an individual tastes something that they see visually, there is a chance the taste will change based on what the individual has registered in the brain. Booth et al emphasize the role of sight: “A mouthful usually stimulates sight first and then touch, taste and smell.” [15] However, there is a possibility that one’s vision may lead to an incorrect interpretation of taste. For example, changing only the color of a drink has a direct effect on its taste [16]. Therefore, changing appearance affects taste based on a person’s visual perception [17]. For example, in one study on changing the color of fruit drinks, color was found to influence sweetness and intensity of a typical flavor [18]. Moir [19] published more than 150 studies examining the influence of vision on taste and flavor. The majority of this research showed that changing the hue and/or intensity of the color added to a food or a beverage can affect the perceived identity and/or intensity of the flavor [19].

Apart from vision, altering touch also affects taste. The touch system contributes to this constancy because taste sensations appear to be localized by touch [20]. Touch, whether by mouth or by hand, has a far greater influence on perceptions of taste, quality, and satiety than we realize [13]. The tip of the tongue is an area of high receptor density, so taste sensation is strongest at the tip but begins to weaken farther back on the tongue [20]. Thus, for years, researchers have investigated how the act of touching products affects consumer response. Phenomena such as the firmness of a cup in which water is served affecting consumers’ judgments of the water’s taste have been shown [17]. The evidence of the effect of vision and touch on perception and taste may affect smokers as well when the external or internal packaging of the cigarette is changed.

### Effect of Prominent Pictorial Warnings

A known sensory factor that influences smokers’ product acceptance and satisfaction is pictorial warnings on tobacco products [21]. The recently implemented plain tobacco packaging regulation in Saudi Arabia was accompanied by the implementation of a new set of pictorial warnings that was more prominent compared with the previously implemented ones. This change was the first in terms of changing pictorial warnings since their introduction in Saudi Arabia in August 2011 [21].

A previous study on a Saudi sample in Saudi Arabia investigated the effect of prominent pictorial warnings compared to the old pictorial warning used before the plain packaging change and showed that the two prominent sets of pictorial warnings scored, on average, 13.1 and 10.2 rating points higher than the old pictorial warnings in increasing participants’ worries on the Brief Worry Scale about Smoking (BWS). The BWS is a 4-item scale that measures worry about physical health as a consequence of smoking [21]. Furthermore, on average, the two prominent sets of pictorial warnings scored 12.5 and 10.1 rating points higher than the old pictorial warnings in increasing participants’ negative reactions on the Self-Assessment Manikin (SAM) [21]. The SAM is a 3-item, nonverbal pictorial assessment method that measures the pleasure, arousal, and dominance associated with a person’s reaction to a wide variety of stimuli [21]. These results indicate the expected stronger emotional evocation in response to the change in pictorial warnings in Saudi Arabia.

As mentioned above, a great deal of scientific research on sensory influences on taste and flavor exists in the food-related domain, but to our knowledge, no research addresses the subject in relation to smoking. Thus, this trial strives to address the following concerns in the most scientific and ethically possible ways: (1) Do smokers who are blinded to cigarette branding report differences in taste between branded and unbranded cigarettes? (2) Do smokers who are blinded to cigarette branding experience differences in immediate coughing between branded and unbranded cigarettes? (3) Do smokers who are blinded to cigarette branding experience differences in shortness of breath between branded and unbranded cigarettes?

In addition, this paper discusses the lesson learned from the Saudi experience with regard to this trial’s findings and other available evidence.

## Methods

### Study Design

A prospective within-person, randomized crossover design was used to address the objectives of this study.

### Exposure and Procedure

Participants received 6 sequences of different random exposures to 3 plain-packaged cigarettes (2 from the favorite brand and 1 from another brand as a control) and 3 branded cigarettes (2 from the favorite brand and 1 from another brand as a control), with a washout period of 5-10 minutes between each cigarette. To ensure reliability and reproducibility of the results, and due to the small sample size, all participants were invited to repeat the trial on another day.

The standard cigarette in Saudi Arabia is approximately 5.5 cm long, excluding the filter. To reduce harm to participants, they were exposed to approximately 3 puffs, which represents around 2 cm of cigarette. To ensure the participants did not exceed this amount, aluminum foil was wrapped around the rest of the cigarette. The use of 3 puffs or 2 cm was determined by the researchers to provide participants with a sufficient opportunity to judge the taste.

To ensure concealment, the cigarettes were provided in random order by a blinding handler who was involved in neither data collection nor data analysis.

**Figure 1:**
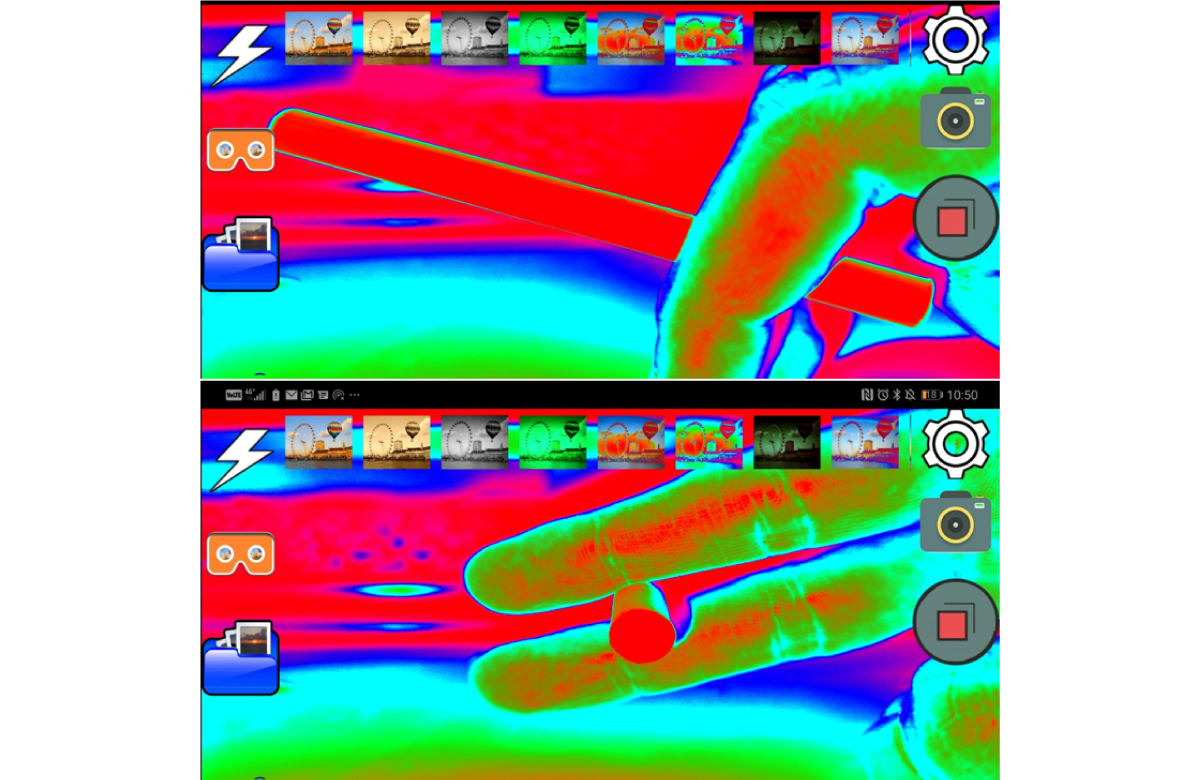
Screenshots of the cigarette blinding app for virtual reality.

To blind participants from recognizing the type of cigarette, each participant wore virtual reality goggles, accompanied by special software to alter the visual reality of the smoker (Figure 1). In addition, the participants wore medical gloves to alter the feeling of touching the cigarettes with their hands.

### Inclusion and Exclusion Criteria

Participants were current cigarette smokers 18 years or older who were cleared upon physical assessment before the experiment.

Candidates who were planning to quit smoking or in a quitting stage were not eligible to participate in this study, thereby reducing harm and ethically avoiding an alteration to their quitting process or plan. Candidates who had respiratory or cardiac disease, a taste-related disorder (eg, hypogeusia or ageusia), or an acute disease that affected taste or smell (eg, flu) were not eligible. Candidates who showed abnormal vital signs, which were checked before the study (fever, shortness of breath, or elevated blood pressure), were not eligible.

### Recruitment

Candidates were invited from the Sharik research participants’ database [22], which includes around 6000 smokers. Candidates received a phone call, and study information and consent were presented to them. If they agreed to participate, then their eligibility conditions were checked. If eligible, then an appointment was booked for them at the study site. Once the participants arrived, the researchers explained the details of the study to them, and written participant information sheets were provided. Participants who wanted to start the study were asked to sign the consent forms. Then, their vital signs were checked. Participant recruitment started in early January 2020 via phone interviews.

### Data Collection and Outcome Measure

#### Demographics and Baseline

Data collection started with a quick interview survey that gathered their age, gender, Fagerstrom Test for Nicotine Dependence score, age they started smoking, and frequency of coughing that lasted 2 weeks.

#### Taste Change

The main outcome measure for the taste was taken by asking the participants to identify the type of cigarette they had smoked (plain or branded) based on taste. In addition, each smoker was asked to rate the taste of the cigarettes on a scale from 1 (*very bad taste*) to 7 (*very good taste*) and the heat perception of the cigarette smoke (burning sensation) from 1 (*acceptable*) to 7 (*unacceptable*). Finally, each smoker was asked whether the cigarette was his or her favorite brand.

#### Cough and Shortness of Breath

The outcome measure for immediate coughing was an observation of any coughing event during the smoking or washout period for each cigarette.

The outcome measure for shortness of breath was measured via self-reporting and peak flow test. We used an approved medical-grade electronic flow test device, following the recommended standard for performing the flow test [23]. Each participant received instructions and performed the flow test accurately before starting the experiment. The participants repeated the test before and after each cigarette for safety reasons. However, the main measure here was the comparison between plain and branded cigarettes because it was delivered in random order for each participant.

Shortness of breath was defined on the peak flow if the reading was 40% less than the baseline before starting the first cigarette because readings of 50% less than the baseline are defined as the signal for medical alert [24].

After completing the experiment, the participants were asked about their perceptions of the taste and health concern claims circulated in the media about plain-packaged tobacco for comparison to their initial opinion before the experiment. The participants were asked, “Do you believe that the taste and content of the new plain-packaged cigarettes have changed compared to the old, branded cigarettes?”

### Sample Size

Based on the smokers’ complaints across media channels, the difference in taste between the plain and branded cigarettes seemed large to medium. Thus, a single-factor, repeated-measures design with a sample of 18 subjects, measured at 6 time points to produce 108 experiments, achieves 80% power to detect a contrast using a multivariate *T*² test at a .05 significance level at 0.45-0.35 effect size [25,26]. Effect size was selected based on Cohen *d* medium effect size due to the lack of sufficient information to calculate a more accurate effect size [27]. Sample size was calculated via PASS 2019, version 19.0.3 (NCSS), using the abovementioned inputs.

### Data Analysis

Descriptive statistics were used to describe the sample demographics, and mixed-model analysis was used to analyze repeated measures of taste-related outcomes to account for within-person differences. A *t* test was used to analyze the ability of participants to identify their favorite brands, and *t* tests were used to analyze the follow-up data due to the small sample size, which prevented the use of repeated-measures mixed-model analysis.

### Ethical Considerations

The study was performed in agreement with the Declaration of Helsinki. The Alfaisal University Institutional Review Board granted ethical approval with approval number IRB-20013. All participants signed the consent form approved by the Alfaisal University Institutional Review Board to participate in this study.

## Results

### Demographics and Baseline

Twenty-five participants were approached; out of these, 1 participant was excluded because he was in a quitting stage, and 2 were excluded for having a cardiovascular disease. Of the eligible participants, 5 did not show up. The 18 participants included 1 woman (6%) and 17 men (94%). Their mean age was 28.9 years (range 19-63), mean nicotine dependency score was 3.3 (range 2-5), and mean number of cigarettes smoked per day was 18.3 (range 12-24). In the prior 2 weeks, 16 out of 18 participants (89%) did not have a coughing attack, and 2 participants had 1 or more coughing attacks.

In terms of participants’ pre-experiment opinions about changes in the new plain-packaged cigarettes’ taste and content, 16 out of 18 participants (89%) thought they detected a change compared to the old branded cigarettes. However, after the experiment, all participants reported that they had changed their opinion and did not believe any differences existed between plain-packaged and branded cigarettes.

### Taste Change

Mixed-model analysis showed no significant differences in participants’ ability (between and within participants) to identify the correct type of cigarettes (plain versus branded cigarettes): the estimate of fixed effect was −0.01 (*P*=.79) (Table 1).

**Table 1:** Summary of the results for the main questions of this study.

Question	Measure	Results	*P* value
Do smokers who are blinded to cigarette branding report differences in taste between branded and unbranded cigarettes?	Participants’ ability to identify plain or branded cigarettes correctly	Estimate of fixed effect=−0.01	.79
Do smokers who are blinded to cigarette branding report differences in taste between branded and unbranded cigarettes?	Taste and burning sensation ratings	Estimate of fixed effect for taste=−0.31; estimate of fixed effect for burning sensation=−0.25	.30; .42
Do smokers who are blinded to cigarette branding report differences in taste between branded and unbranded cigarettes?	Participants’ ability to identify their favorite brand compared to other brands used in the experiment	*t*_107_=−0.63, mean 0.47	.53
Do smokers who are blinded to cigarette branding experience differences in immediate coughing between branded and unbranded cigarettes?	Coughing incidents	Out of the 108 experiments, 1 incident of short coughing was observed	N/A^a^
Do smokers who are blinded to cigarette branding have differences in shortness of breath between branded and unbranded cigarettes?	Shortness of breath incidents	Out of the 108 experiments, 0 incidents of short coughing were observed	N/A

^a^N/A: not applicable.

In terms of taste and burning sensation ratings, no significant differences were observed in the rating scores between the plain and the branded cigarettes, between and within participants: the estimate of fixed effect for taste was −0.31 (*P*=.30), and the estimate of fixed effect for burning sensation was −0.25 (*P*=.42).

Finally, no differences were seen in the same participants’ ability to identify their favorite brand versus another nonfavorite brand (*t*_107_=−0.63, mean 0.47, *P*=.53).

### Cough and Shortness of Breath

In terms of immediate coughing, out of the 108 experiments, 1 short coughing episode was observed, which was attributed to a branded cigarette, not a plain-packaged cigarette.

None of the participants in the 108 experiments reported shortness of breath. In addition, comparing the changes in peak flow reading between the first cigarettes, none of the participants had shortness of breath. Finally, no cases of shortness of breath were recorded overall after the full experiment for all participants.

## Discussion

### Summary of Findings

This study investigated the claims of taste change, immediate continuous coughing, and immediate shortness of breath allegedly caused by plain-packaged cigarettes. After controlling participants’ visual and touch perceptions, no significant differences were observed in their ability to identify plain versus branded cigarettes, and more surprisingly, no significant differences were seen in their ability to identify their favorite brand versus nonfavorite brands. No alarming findings emerged related to immediate cough or shortness of breath. Most of the study participants were men, because the prevalence of women smokers is very low (1.5%) compared to men smokers (26.2%) in Saudi Arabia [1,2].

### Summary of the Issue Escalation

Smokers in Saudi Arabia have complained about the look, feel, and taste of the newly introduced plain-packaged cigarettes. This was also made worse by introducing the new and emotionally evocative pictorial warnings at the same time, which proved to signal large health concerns in the same population in a previous study [21].

Moreover, another issue in the Saudi implementation process was the lack of a public awareness campaign before implementation. Without prior notice, the arrival of new cigarette packaging and emotionally evocative pictorial warnings sent smokers a shock, which could have directed them to seek answers and create theories.

In addition, after the issue had escalated, the authorities lightly explained that the plain packaging was a new regulation, but this explanation only addressed the outer packaging, not the internal (paper and filter) changes or the new pictorial warning [7,8,28]. This response left the majority with unanswered questions and concerns.

Although tobacco companies then released statements (Multimedia Appendix 1) declaring no changes in the cigarette content except what was required by the new regulation, they did not provide consumers with details of the changes called for in the new regulation; thus, the dilemma was dragged out. Later, the authorities revised the plain-packaging standards to allow tobacco companies to reintroduce cigarettes with their original branded (paper and filter) look, while keeping the external plain packaging, which was a victory for tobacco companies. This change is a reduction of the World Health Organization plain-packaging standard and may reduce the effectiveness and benefits of the plain-packaging strategy.

Finally, it is worth noting that sensory perception and sensory research are priorities within the tobacco industry because they have direct effects on commercial concerns [29]. Sensory aspects contribute to smoker satisfaction and tobacco product acceptance, and they play an important role in controlling cigarette-puffing behavior. Tobacco companies have capitalized on distinct sensory preferences across gender, age, and ethnic groups by tailoring products for specific populations [29]. This study provided evidence that with the use of virtual reality and gloves to blind participants, they were not able to differentiate between their favorite brand and other nonfavorite brands. This highlights the fact that the tobacco industry understands such a topic in more depth and detail than regulators generally do.

Overall, this issue has some important lessons to be considered in any future tobacco policy changes.

### Reorganizing the Chain of Events and Lessons Learned

The implementation of plain packaging in Saudi Arabia has represented a major chain of decisions that led to the current situation, which almost ruined the implementation of an effective public health policy. The major factors are as follows: (1) lack of a preimplementation awareness campaign, which played a major role in convincing the consumers that the plain-packaged cigarettes were counterfeit; (2) introducing the new pictorial warnings at the same time, having previous knowledge that it could cause consumer anxiety, with no preimplementation awareness campaign of this change either; (3) lack of prior risk assessment of the potential negative effects of such implementation on stakeholders; (4) lack of awareness about the plain-packaging standards, including explanation of why the externals and internals of the products had changed and a delay in the response to consumers’ concerns, especially health-related ones; and (5) lack of scientifically sound evidence to explain the claims of taste changes and health concerns.

The most prominent factor here was the lack of proper risk assessment before implementation, which could have highlighted the potential risks before the implementation and improved the decision-making process. The impact of the new changes was a life change for a smoker unused to such strong public health actions. As one of the study participants described it, “I woke up one day and found that all cigarettes brands have the same look and feel. [I thought] they must be counterfeit, there was no other explanation.”

The mode of implementation also underestimated the role of social media in spreading fear and rumors to counteract the implementation of the new regulation, especially with the known history of how tobacco industries strive to undermine tobacco control regulations [30,31]. The claims about the plain packaging, including taste and quality, have been raised in other countries that implemented plain-packaging regulations, such as Australia and the United Kingdom [6,9,10], and the assumption that it was predictable for such claims to be reused in counter-policy campaigns was also overlooked.

Instead of being proactive with the consumers and engaging with them to explain the new regulation, the authorities maintained their silence for a long time, with a few contradicting announcements that started by denying any changes in physical and chemical content, then confirming the changes in quality and taste and blaming tobacco companies [7,28]. Tobacco companies, in return, stated they had made no changes. As a result, consumers did not know what had changed, how, or why. None of the statements by authorities or tobacco companies explained to consumers why all cigarettes looked the same (paper and filter) or why packets had new and emotionally provocative pictorial warnings.

Unfortunately, the same repellent effects (evoking smokers’ health concerns and removing the appeal and charm of brands) that reflected the desire to encourage smokers to quit and to prevent nonsmokers from smoking, when introduced in this order and rushed into implementation, caused unexpected negative effects that neutralized the policy.

### Conclusion

This study afforded a new dimension in understanding why some smokers find the taste of plain-packaged cigarettes differs from the branded cigarettes. After controlling the visual and touch sensations, participants were not able to differentiate between branded and plain-packaged cigarettes in terms of taste or inducing immediate shortness of breath or cough. Interestingly, participants were not able to identify their favorite brand.

## Appendix

Multimedia Appendix 1 Tobacco companies' public statements declaring no changes to their products' content.

## Abbreviations

BWS: Brief Worry Scale about Smoking
SAM: Self-Assessment Manikin
