# Supplementary material for: Reporting of Differences in Taste Between Branded and Unbranded Cigarettes by Smokers Blinded to Cigarette Branding: Within-Person, Randomized Crossover Study
Source: JMIR Form Res. 2021 May 14;5(5):e24446. doi: 10.2196/24446 (PMC8164127; doi:10.2196/24446)
Supplement: Multimedia Appendix 1 [file formative_v5i5e24446_app1.pdf]

British American Tobacco
ME DMCC
37th floor, JBC 3
Cluster Y, JLT
P.O. Box 337222, Dubai
United Arab Emirates

Tel +971 4 365 9500
Fax +971 4 369 7404

بريتش أميركان توباكو
إم إي م.د.م.س
طابق ٣٧ برج ال JBC 3
المجمع Y
أبراج بحيرة الجميرا
ص.ب. 337222، دبي
الإمارات العربية المتحدة

هاتف 9500 365 4 971+
فاكس 7404 369 4 971+
www.batme.com

British American Tobacco (BAT) is aware of recent consumer social media posts questioning the legitimacy of various cigarette products following the implementation of plain packaging in Saudi Arabia (KSA).

علمت شركة بريتش أميركان توباكو بمنشورات يتم تداولها على مواقع التواصل الاجتماعي تشكك بشرعية مختلف منتجات التبغ بعد تطبيق التغليف العادي في المملكة العربية السعودية.

BAT, manufacturer of cigarette brands around the world including Pall Mall, Dunhill, JPGL, Rothmans, Vogue and Kent, complies with all laws, regulations and standards within all countries it sells its products.

ان شركة بريتش أميركان توباكو المصنعة لأصناف السجائر في العالم ومن ضمنها Pall Mall، Dunhill، JPGL، Rothmans، Vogue وKent، تلتزم بجميع القوانين والأنظمة واللوائح في جميع البلاد التي تبيع فيها منتجاتها.

The Saudi Food & Drug Authority (SFDA) had previously issued the Plain Packaging standard (SFDA.FD 60:2018) which requires all tobacco products imported into KSA from 23 August 2019 to have standardised plain packaging. BAT is complying with this SFDA standard.

أصدرت هيئة الغذاء والدواء السعودية لائحة التغليف العادي (SFDA.FD 60:2018) التي تفرض اعتماد التغليف العادي الموحد على كل منتجات التبغ المستوردة الى المملكة العربية السعودية بعد تاريخ ٢٣ أغسطس ٢٠١٩. والتزمت شركة بريتش أميركان توباكو بلائحة هيئة الغذاء والدواء السعودية هذه.

The main requirements of the standard are:

- Standardised packaging across all brands is required with no colours and designs. The only allowed differentiation is the brand and type name, and the manufacturer's details. All other information, including date of manufacturing and deliveries (tar, nicotine and CO) are not allowed;
- Only full plain white cigarettes are allowed, with no designs.

ان المتطلبات الأساسية لهذه اللائحة هي:

- تغليف موحد لكل الأصناف دون استعمال ألوان أو تصاميم. ان التفرقة الوحيدة المسموحة هي باسم العلامة والنوع، وتفاصيل المصنع. ولا يسمح بذكر باقي المعلومات كتاريخ الانتاج والاصدارات (قطران، نيكوتين و أول أوكسيد الكاربون)،
- يسمح بالسجائر المغلفة بالأبيض فقط دون أي تصميم.

Incorporated as a Free Zone Limited Liability Company under Dubai Multi Commodity Centre Rules & Regulations
تأسست كشركة منطقة حرة ذات مسؤولية محدودة ضمن قواعد ولوائح مركز دبي للسلع المتعددة

BRITISH AMERICAN
TOBACCO
MIDDLE EAST

British American Tobacco
ME DMCC
37th floor, JBC 3
Cluster Y, JLT
P.O. Box 337222, Dubai
United Arab Emirates

Tel +971 4 365 9500
Fax +971 4 369 7404

بريتيش أميركان توباكو
إم إي م.د.م.س
طابق ٣٧ برج ال JBC 3
المجمع Y
أبراج بحيرة الجميرا
ص.ب. 337222، دبي
الإمارات العربية المتحدة

هاتف +971 4 365 9500
فاكس +971 4 369 7404
www.batme.com

However, as far as BAT cigarette brands are concerned, the tobacco blend used remains exactly as it was pre-plain packaging, delivering the same flavour to the consumer. BAT continues to be committed to providing consumers with high standard, quality products, complying with all laws and regulations.

وبما يتعلق بأصناف سجائر شركة بريتش أميركان توباكو، ان خلطة التبغ المستعملة هي نفسها كما كانت عليه قبل تطبيق التغليف العادي، مع توفير النكهة ذاتها للمستهلك. ان شركة بريتش أميركان توباكو تستمر بالالتزام بتوفير للمستهلكين بمنتجات عالية الجودة والمعايير، وبجميع القوانين والأنظمة.

If you have any questions on BAT brands, please contact freephone 8008971442.

للاستفسار حول أي من منتجات بريتش أميركان توباكو، نرجو الاتصال على الرقم المجاني 8008971442.

بريتيش أميركان توباكو إم إي م.د.م.س
BRITISH AMERICAN
TOBACCO
ME DMCC
PO BOX 337222 DUBAI UAE
TRN 100020546600003

Incorporated as a Free Zone Limited Liability Company under Dubai Multi Commodity Centre Rules & Regulations
تأسست كشركة منطقة حرة ذات مسؤولية محدودة ضمن قواعد ولوائح مركز دبي للسلع المتعددة
